# Supplementary material for: Health and economic impact of rotavirus vaccination in GAVI-eligible countries
Source: BMC Public Health. 2010 May 14;10:253. doi: 10.1186/1471-2458-10-253 (PMC2893091; doi:10.1186/1471-2458-10-253)
Supplement: Additional file 1 — Supplementary information on model inputs and results. The document provides detailed information on some of the key model inputs and results, including vaccine efficacy adjusted for serotype distributions, length of stay for rotavirus associated hospitalizations, hourly wage, transportation costs, and country-specific budget impact analysis results. [file 1471-2458-10-253-S1.DOC]

**Health and economic impact of rotavirus vaccination in GAVI-eligible countries**

Sun-Young Kim, Steve Sweet, David Slichter, Sue J. Goldie

**Supplementary information on model inputs and results**

**Appendix 1. Rotavirus serotype distributions and vaccine efficacy adjusted for serotypes**

We searched for studies on serotype distribution with an English-language search on PubMed. Search terms included “rotavirus”, “serotype”, “genotype”, “strain”, and the names of individual countries. We took the studies most likely to be accurate – most recent, and with largest sample size – as our base case, and used other available studies to estimate error.

Serotype-specific vaccine efficacy data were obtained from published clinical trial studies. Mean vaccine efficacy against each serotype is as follows:

- G1P[8]: 87.43%

- G3P[8]: 90.19%

- G4P[8]: 93.37%

- G9P[8]: 83.76%

- G2P[4], other combination of G and P: 71.42%

| Country | G1P[8] | G3P[8] | G4P[8] | G9P[8] | G2P[4], other combination of G and P | Vaccine efficacy adj. for serotype distribution | Vaccine efficacy (SAGE approach) |
| --- | --- | --- | --- | --- | --- | --- | --- |
| Afghanistan | 0.17 | 0.01 | 0.03 | 0.05 | 0.74 | 75.6% | 49.5% |
| Angola | 0.36 | 0.00 | 0.00 | 0.10 | 0.54 | 78.4% | 49.5% |
| Armenia | 0.32 | 0.04 | 0.18 | 0.20 | 0.26 | 83.7% | 76.9% |
| Azerbaijan | 0.32 | 0.04 | 0.18 | 0.20 | 0.26 | 83.7% | 49.5% |
| Bangladesh | 0.12 | 0.03 | 0.17 | 0.12 | 0.57 | 78.9% | 76.9% |
| Benin | 0.36 | 0.00 | 0.00 | 0.10 | 0.54 | 78.4% | 49.5% |
| Bhutan | 0.21 | 0.14 | 0.04 | 0.29 | 0.32 | 81.9% | 76.9% |
| Bolivia | 0.32 | 0.03 | 0.05 | 0.21 | 0.39 | 80.8% | 76.9% |
| Burkina Faso | 0.02 | 0.00 | 0.00 | 0.00 | 0.98 | 71.8% | 49.5% |
| Burundi | 0.36 | 0.00 | 0.00 | 0.10 | 0.54 | 78.4% | 49.5% |
| Cambodia | 0.21 | 0.14 | 0.04 | 0.29 | 0.32 | 81.9% | 49.5% |
| Cameroon | 0.21 | 0.13 | 0.02 | 0.00 | 0.64 | 77.6% | 49.5% |
| Central African Republic | 0.36 | 0.00 | 0.00 | 0.10 | 0.54 | 78.4% | 49.5% |
| Chad | 0.36 | 0.00 | 0.00 | 0.10 | 0.54 | 78.4% | 49.5% |
| Comoros | 0.36 | 0.00 | 0.00 | 0.10 | 0.54 | 78.4% | 76.9% |
| Congo | 0.36 | 0.00 | 0.00 | 0.10 | 0.54 | 78.4% | 49.5% |
| Cote d'Ivoire | 0.09 | 0.00 | 0.00 | 0.00 | 0.91 | 72.8% | 49.5% |
| Cuba | 0.32 | 0.03 | 0.05 | 0.21 | 0.39 | 80.8% | 76.9% |
| Democratic Republic of the Congo | 0.36 | 0.00 | 0.00 | 0.10 | 0.54 | 78.4% | 49.5% |
| Djibouti | 0.17 | 0.01 | 0.03 | 0.05 | 0.74 | 75.6% | 49.5% |
| Eritrea | 0.36 | 0.00 | 0.00 | 0.10 | 0.54 | 78.4% | 76.9% |
| Ethiopia | 0.36 | 0.00 | 0.00 | 0.10 | 0.54 | 78.4% | 49.5% |
| Georgia | 0.32 | 0.04 | 0.18 | 0.20 | 0.26 | 83.7% | 76.9% |
| Ghana | 0.80 | 0.00 | 0.00 | 0.04 | 0.17 | 84.7% | 49.5% |
| Guinea | 0.36 | 0.00 | 0.00 | 0.10 | 0.54 | 78.4% | 49.5% |
| Guinea-Bissau | 0.03 | 0.00 | 0.00 | 0.00 | 0.97 | 71.9% | 49.5% |
| Guyana | 0.32 | 0.03 | 0.05 | 0.21 | 0.39 | 80.8% | 76.9% |
| Haiti | 0.32 | 0.03 | 0.05 | 0.21 | 0.39 | 80.8% | 49.5% |
| Honduras | 0.32 | 0.03 | 0.05 | 0.21 | 0.39 | 80.8% | 76.9% |
| India | 0.24 | 0.00 | 0.00 | 0.04 | 0.72 | 75.8% | 76.9% |
| Indonesia | 0.21 | 0.14 | 0.04 | 0.29 | 0.32 | 81.9% | 76.9% |
| Kenya | 0.36 | 0.00 | 0.00 | 0.10 | 0.54 | 78.4% | 49.5% |
| Kiribati | 0.21 | 0.14 | 0.04 | 0.29 | 0.32 | 81.9% | 76.9% |
| Korea, Democratic Republic | 0.21 | 0.14 | 0.04 | 0.29 | 0.32 | 81.9% | 76.9% |
| Kyrgyzstan | 0.32 | 0.04 | 0.18 | 0.20 | 0.26 | 83.7% | 76.9% |
| Lao People Democratic Republic | 0.21 | 0.14 | 0.04 | 0.29 | 0.32 | 81.9% | 76.9% |
| Lesotho | 0.36 | 0.00 | 0.00 | 0.10 | 0.54 | 78.4% | 49.5% |
| Liberia | 0.36 | 0.00 | 0.00 | 0.10 | 0.54 | 78.4% | 49.5% |
| Madagascar | 0.36 | 0.00 | 0.00 | 0.10 | 0.54 | 78.4% | 49.5% |
| Malawi | 0.27 | 0.22 | 0.05 | 0.00 | 0.46 | 81.0% | 49.5% |
| Mali | 0.36 | 0.00 | 0.00 | 0.10 | 0.54 | 78.4% | 49.5% |
| Mauritania | 0.36 | 0.00 | 0.00 | 0.10 | 0.54 | 78.4% | 49.5% |
| Moldova | 0.32 | 0.04 | 0.18 | 0.20 | 0.26 | 83.7% | 76.9% |
| Mongolia | 0.21 | 0.14 | 0.04 | 0.29 | 0.32 | 81.9% | 76.9% |
| Mozambique | 0.36 | 0.00 | 0.00 | 0.10 | 0.54 | 78.4% | 49.5% |
| Myanmar | 0.21 | 0.14 | 0.04 | 0.29 | 0.32 | 81.9% | 49.5% |
| Nepal | 0.71 | 0.00 | 0.00 | 0.00 | 0.29 | 82.8% | 76.9% |
| Nicaragua | 0.40 | 0.20 | 0.00 | 0.00 | 0.41 | 81.4% | 76.9% |
| Niger | 0.36 | 0.00 | 0.00 | 0.10 | 0.54 | 78.4% | 49.5% |
| Nigeria | 0.09 | 0.00 | 0.00 | 0.00 | 0.91 | 72.9% | 49.5% |
| Pakistan | 0.17 | 0.01 | 0.03 | 0.05 | 0.74 | 75.6% | 49.5% |
| Papua New Guinea | 0.21 | 0.14 | 0.04 | 0.29 | 0.32 | 81.9% | 76.9% |
| Rwanda | 0.36 | 0.00 | 0.00 | 0.10 | 0.54 | 78.4% | 49.5% |
| Sao Thome | 0.36 | 0.00 | 0.00 | 0.10 | 0.54 | 78.4% | 49.5% |
| Senegal | 0.36 | 0.00 | 0.00 | 0.10 | 0.54 | 78.4% | 49.5% |
| Sierra Leone | 0.36 | 0.00 | 0.00 | 0.10 | 0.54 | 78.4% | 49.5% |
| Solomon Islands | 0.21 | 0.14 | 0.04 | 0.29 | 0.32 | 81.9% | 76.9% |
| Somalia | 0.36 | 0.00 | 0.00 | 0.10 | 0.54 | 78.4% | 49.5% |
| Sri Lanka | 0.21 | 0.14 | 0.04 | 0.29 | 0.32 | 81.9% | 76.9% |
| Sudan | 0.17 | 0.01 | 0.03 | 0.05 | 0.74 | 75.6% | 49.5% |
| Tajikistan | 0.32 | 0.04 | 0.18 | 0.20 | 0.26 | 83.7% | 76.9% |
| Tanzania | 0.09 | 0.00 | 0.00 | 0.68 | 0.23 | 81.2% | 49.5% |
| The Gambia | 0.36 | 0.00 | 0.00 | 0.10 | 0.54 | 78.4% | 49.5% |
| Timor Leste | 0.21 | 0.14 | 0.04 | 0.29 | 0.32 | 81.9% | 76.9% |
| Togo | 0.36 | 0.00 | 0.00 | 0.10 | 0.54 | 78.4% | 49.5% |
| Uganda | 0.36 | 0.00 | 0.00 | 0.10 | 0.54 | 78.4% | 49.5% |
| Ukraine | 0.32 | 0.04 | 0.18 | 0.20 | 0.26 | 83.7% | 76.9% |
| Uzbekistan | 0.32 | 0.04 | 0.18 | 0.20 | 0.26 | 83.7% | 76.9% |
| Viet Nam | 0.45 | 0.26 |  |  | 0.29 | 83.5% | 76.9% |
| Yemen | 0.17 | 0.01 | 0.03 | 0.05 | 0.74 | 75.6% | 49.5% |
| Zambia | 0.09 | 0.02 | 0.00 | 0.00 | 0.89 | 73.2% | 49.5% |
| Zimbabwe | 0.03 | 0.09 | 0.00 | 0.00 | 0.88 | 73.6% | 49.5% |

**References**

Dey SK, Hayakawa Y, Rahman M, Islam R, Mizuguchi M, Okitsu S, Ushijima H. G2 strain of rotavirus among infants and children, Bangladesh. *Emerg Infect Dis* 2009; 15(1):91-4.

Steele AD, Ivanoff B. Rotavirus strains circulating in Africa during 1996-1999: emergence of G9 strains and P[6] strains. *Vaccine* 2003; 21:361-7.

Silva PA, Stark K, Mockenhaupt FP, Reither K, Weitzel T, Ignatius R et al. Molecular characterization of enteric viral agents from children in northern region of Ghana. *J Med Virol* 2008; 80(10):1790-8.

Nielsen NM, Eugen-Olsen J, Aaby P, Molbak K, Rodrigues A, Fischer TK. Characterisation of rotavirus strains among hospitalized and non-hospitalised children in Guinea-Bissau, 2002: A high frequency of mixed infections with serotype G8. *J Clin Virol* 2005; 34(1):13-21.

Samajdar S, Ghosh S, Chawla-Sarkar M, Mitra U, Dutta P, Kobayashi N et al. Increase in prevalence of human group A rotavirus G9 strains as an important VP7 genotype among children in eastern India. *J Clin Virol* 2008; 43(3):334-9.

Banerjee I, Ramani S, Primrose B, Moses P, Iturriza-Gomara M, Gray JJ et al. Comparative study of the epidemiology of rotavirus in children from a community-based birth cohort and a hospital in South India. *J Clin Microbiol* 2006; 44(7):2468-74.

Gentsch JR, Laird AR, Bielfelt B, Griffin DD, Banyai K, Ramachandran M et al. Serotype diversity and reassortment between human and animal rotavirus strains: implications for rotavirus vaccine programs. *J Infect Dis* 2005; 192(Suppl 1):S146-59.

Uchida R, Pandey BD, Sherchand JB, Ahmed K, Yokoo M, Nakagomi T et al. Molecular epidemiology of rotavirus diarrhea among children and adults in Nepal: detection of G12 strains with P[6] or P[8] and a G11P[25] strain. *J Clin Microbiol* 2006; 44(10):3499-505.

Espinoza F, Bucardo F, Paniagua M, Svensson L, Hallander HO, Bondeson K. Shifts of rotavirus g and p types in Nicaragua—2001-2003. *Pediatr Infect Dis J* 2006; 25(11):1078-80.

Moyo SJ, Gro N, Kirsti V, Matee MI, Kitundu J, Maselle SY et al. Prevalence of enteropathogenic viruses and molecular characterization of group A rotavirus among children with diarrhea in Dar es Salaam Tanzania. *BMC Public Health* 2007; 7:359.

**Appendix 2. Estimated length of stay**

There is a very limited amount of literature on average length of stay for children hospitalized with rotavirus. Mendelsohn *et al.* (2008) gives average length of stay for two hospitals in India as 2 and 3 days, but the sample sizes were small; Nokes *et al.* (2008) gives an average stay of 5.2 days at a hospital in Kenya; Fischer (2005) gives stays of 4.3 and 4.8 for an urban and a rural hospital in Vietnam, respectively; Isakbaeva (2007) finds an average stay of 6.8 days in Uzbekistan; Nielsen (2005) finds an average stay of 9.5 days in Guinea-Bissau; Ehrenkranz (2001) finds an average stay of 1.1 days at a hospital in Peru; Podewils (2005) uses a base case estimate of 4.5 days in Asia; and Rheingans (2005) uses an estimate of 3.5 days across Latin America. From this set of data, we chose to estimate the average stay at 3 days.

**References**

Mendelsohn AS, Asirvatham JR, Mwamburi D, Sowmynarayanan TV, Mlalik V, Muliyil J et al. Estimate of the economic burden of rotavirus-associated and all-cause diarrhoea in Vellore, India. *Trop Med Int Health* 2008, 13(7):934-42.

Nokes DJ, Abwao J, Pamba A, Peenze I, Dewar J, Maghenda JK et al. Incidence and clinical characteristic of group A rotavirus infections among children admitted to hospital in Kilifi, Kenya. *PLos Med* 2008; 5(7):e153.

Fischer TK, Anh DD, Antil L, Cat ND, Kilgore PM, Thiem VD, Rheingans R, Tho le H, Glass RI, Bresee JS. Health care costs of diarrheal disease and estimates of the cost-effectiveness of rotavirus vaccination in Vietnam. *J Infect Dis* 2005; 192(10):1720-6.

Isakbaeva ET, Musabaev E, Antil L, Rheingans R, Juraev R, Glass RI et al. Rotavirus disease in Uzbekistan: Cost-effectiveness of a new vaccine. *Vaccine* 2007; 25(2):373-80.

Nielsen NM, Eugen-Olsen J, Aaby P, Molbak K, Rodrigues A, Fischer TK. Characterisation of rotavirus strains among hospitalized and non-hospitalised children in Guinea-Bissau, 2002: A high frequency of mixed infections with serotype G8. *J Clin Virol* 2005; 34(1):13-21.

Ehrenkranz P, Lanata CF, Penny ME, Salazar-Lindo E, Glass RI. Rotavirus diarrhea disease burden in Peru: the need for a rotavirus vaccine and its potential cost savings. Rev *Panam Salud Publica* 2001; 10(4):240-8.

Podewils LJ, Antil L, Hummelman E, Bresee J, Parashar UD, Rheingans R. Projected cost-effectiveness of rotavirus vaccination for children in Asia. *J Infect Dis* 2005;Suppl 1:S133-45.

Rheingans RD, Constenia D, Antil L, Innis BL, Breuer T. Potential cost-effectiveness of vaccination for rotavirus gastroenteritis in eight Latin American and Caribbean countries. *Rev Panam Salud Publica* 2007; 21(4):205-16.

**Appendix 3. Country-specific estimation of hourly wage**

We used the ILO’s LABORSTA database (http://laborsta.ilo.org/) to estimate hourly wages. We primarily used the ILO’s database listing wages by specific occupation, in combination with information gleaned from the CIA’s online Factbook (<https://www.cia.gov/library/publications/the-world-factbook/>).

For each country, we started by looking at the Factbook’s breakdown of labor force by occupation. If >60% of the labor force was in agriculture, then we assumed that wages for field workers are the best initial estimate of the relevant wages for our study, and used the field worker wages listed by the ILO. If a breakdown of the labor force was not available, we looked at the breakdown of GDP by sector; to estimate the fraction of labor force involved in agriculture, we multiplied the % of GDP derived from agriculture by 1.5. We used this adjustment because industry and services usually account for a disproportionately large fraction of GDP. Additionally, in some cases, wages were available from the ILO not as “average” but as “minimum” or “maximum” estimates. In cases where only a minimum estimate is available, we adjusted upwards by 20% to reflect the typical spread where multiple rates are quoted.

If <60% of the labor force is employed in agriculture, then we adjusted the estimated wages of agricultural workers using data on service sector employees, who, with agriculture, combine to form over 70% of the work force in all the GAVI countries. Our estimate for service sector employees was calculated as the average wage listed for retail grocery salespeople, waiters, cooks, bus drivers, and refuse collectors. Note that, in instances where service wages were used in estimating hourly wages and a precise breakdown of labor force by sector was not available, we multiplied fraction of GDP by .67 to estimate the fraction of the labor force involved in service work.

We then averaged the service and agricultural wage estimates according to their relative prevalence in the working population. We then multiplied that total by (1 - unemployment rate), with unemployment figures pulled from the CIA Factbook. If the unemployment rate was mixed with the underemployment rate in the Factbook, then we divided the total figure by 2 for our estimate of the unemployment rate. If unemployment rate was not listed, we assumed 10%.

For some countries, data was not available from the ILO’s individual occupational wage database. In those instances, we used estimates for occupational clusters from ILO LABORSTA’s 5a database.

If the ILO had no available data at all for a nation, we estimated wages by averaging our wage estimates in INT$ and US$ across the nation’s WHO group, and multiplied those numbers by the ratio of the target country’s GDP per capita (from the CIA Factbook) to the average GDP per capita in the WHO group.

Finally, for North Korea, no ILO wages were available, and it was decided that it would be inappropriate to extrapolate wage data from other countries.

| Country | Currency: 2005 International dollars | | |
| --- | --- | --- | --- |
|  | Baseline | Low | High |
| Afghanistan | 0.44 | 0.13 | 0.74 |
| Angola | 0.63 | 0.189 | 1.071 |
| Armenia | 1.55 | 0.62 | 2.17 |
| Azerbaijan | 2.05 | 1.03 | 3.08 |
| Bangladesh | 0.33 | 0.17 | 0.5 |
| Benin | 1.49 | 0.75 | 2.24 |
| Bhutan | 0.82 | 0.25 | 1.39 |
| Bolivia | 2.82 | 1.81 | 4.23 |
| Burkina Faso | 0.22 | 0.33 | 0.11 |
| Burundi | 0.19 | 0.29 | 0.1 |
| Cambodia | 0.16 | 0.24 | 0.08 |
| Cameroon | 1.26 | 1.89 | 0.63 |
| Central African Republic | 0.29 | 0.44 | 0.15 |
| Chad | 2.6 | 3.9 | 1.3 |
| Comoros | 1.14 | 1.71 | 0.57 |
| Congo | 1.59 | 2.7 | 0.48 |
| Cote d'Ivoire | 0.33 | 0.5 | 0.17 |
| Cuba | 1.83 | 0.549 | 3.111 |
| Democratic Republic of the Congo | 0.13 | 0.21 | 0.04 |
| Djibouti | 2.58 | 3.87 | 1.29 |
| Eritrea | 0.69 | 1.04 | 0.35 |
| Ethiopia | 0.89 | 1.34 | 0.45 |
| Georgia | 1.13 | 1.58 | 0.45 |
| Ghana | 0.61 | 0.183 | 1.037 |
| Guinea | 0.93 | 0.279 | 1.581 |
| Guinea-Bissau | 0.54 | 0.92 | 0.16 |
| Guyana | 1.07 | 1.61 | 0.54 |
| Haiti | 1.14 | 1.71 | 0.57 |
| Honduras | 1.26 | 1.89 | 0.63 |
| India | 0.22 | 0.33 | 0.11 |
| Indonesia | 0.58 | 0.87 | 0.29 |
| Kenya | 0.45 | 0.68 | 0.23 |
| Kiribati | 1.55 | 2.63 | 0.46 |
| Korea, Democratic Republic | 0.52 | 0.94 | 0.1 |
| Kyrgyzstan | 0.21 | 0.32 | 0.11 |
| Lao People Democratic Republic | 1.02 | 1.73 | 0.31 |
| Lesotho | 0.9 | 1.35 | 0.45 |
| Liberia | 0.14 | 0.21 | 0.07 |
| Madagascar | 3.24 | 4.86 | 1.62 |
| Malawi | 0.44 | 0.66 | 0.22 |
| Mali | 1.05 | 1.58 | 0.53 |
| Mauritania | 1.9 | 3.22 | 0.57 |
| Moldova | 1.1 | 1.65 | 0.55 |
| Mongolia | 0.38 | 0.53 | 0.15 |
| Mozambique | 0.46 | 0.138 | 0.782 |
| Myanmar | 0.19 | 0.057 | 0.323 |
| Nepal | 0.37 | 0.56 | 0.19 |
| Nicaragua | 1.54 | 2.31 | 0.77 |
| Niger | 1.8 | 2.7 | 0.9 |
| Nigeria | 0.27 | 0.41 | 0.14 |
| Pakistan | 0.95 | 1.43 | 0.48 |
| Papua New Guinea | 2.03 | 3.05 | 1.02 |
| Rwanda | 0.47 | 0.71 | 0.24 |
| Sao Thome | 1.17 | 2.00 | 0.35 |
| Senegal | 1.12 | 1.68 | 0.56 |
| Sierra Leone | 0.25 | 0.38 | 0.13 |
| Solomon Islands | 0.86 | 1.29 | 0.43 |
| Somalia | 0.33 | 0.56 | 0.1 |
| Sri Lanka | 0.84 | 1.18 | 0.34 |
| Sudan | 0.07 | 0.021 | 0.119 |
| Tajikistan | 0.1 | 0.03 | 0.17 |
| Tanzania | 0.54 | 0.92 | 0.16 |
| The Gambia | 1.17 | 2 | 0.35 |
| Timor Leste | 0.36 | 0.61 | 0.11 |
| Togo | 0.77 | 1.16 | 0.39 |
| Uganda | 0.16 | 0.24 | 0.08 |
| Ukraine | 1.11 | 1.67 | 0.56 |
| Uzbekistan | 0.2 | 0.06 | 0.34 |
| Viet Nam | 1.36 | 2.3 | 0.41 |
| Yemen | 1.39 | 2.09 | 0.7 |
| Zambia | 0.21 | 0.32 | 0.11 |
| Zimbabwe | 0.08 | 0.14 | 0.03 |

**References**

International Labour Office. Database on labour statistics (LABORSTA). [http://laborsta.ilo.org/].

CIA. The World Factbook (https://www.cia.gov/library/publications/the-world-factbook/countrylisting.html).

**Appendix 4. Household-level average travel costs per visit at medical facilities**

Literature Search

We performed search through PubMed for articles that pertain to “transport* cost” and “travel cost”. This returned a list of around 1300 articles.

First, we read through the titles and pick out ones that pertain to the countries of interest, or have some relevance to the topic of transportation cost and not public maintenance. If the title is ambiguous include it. This left approximately 200 articles.

Next, we read the abstracts of the articles to filter them yet again for discussions of studies regarding or relating to transportation costs. This left approximately 40 articles.

Third, we read through the articles to find transportation costs pertaining to the appropriate countries. This left fourteen articles.

Data Extraction

Some of the articles do not mention transportation cost directly. Accordingly, we made some specific assumptions for each country as follows:

- 1. Bangladesh – Ashworth et al. describe three sets of treatments given: inpatient, day care and domiciliary care. We assumed that day care transportation costs would be similar to a single visit’s transportation costs and thus used those numbers.
  2. Ethiopia – Deressa et al. gives the average for transportation costs for patients visiting first-providers
  3. Ghana – Buor gives the average transportation cost.
  4. Haiti – Mukherjee et al. list transportation fees for monthly clinic visit at $60 per Patient per Year. Assuming only one visit per month, we calculated $5 / visit.
  5. Honduras – Rheingans et al list the transportation costs for outpatient visits.
  6. Malawi – Kemp et al. tables the mean transportation cost for smear-negative patients at MK 317, with an average of 5.9 visits, and MK 222 for smear-positive patients with an average of 4.5 visits. We found the cost per patient per visit and then averaged between the two groups.
  7. Pakistan – Hussain et al. gives the average household cost per health facility visit
  8. Sri Lanka – Attanayake et al. gives the patient’s average cost per visit, informal and formal
  9. Sudan – Gerstl et al. provided a table of data with regards to transportation costs in dry and rainy seasons for three regions. We found a weighted average of the transportation costs across all regions year round, assuming that the dry season and rainy season were both six months long.
  10. Tanzania – Wyss et al. has the transportation costs within the last week per patient. We used that number.
  11. Zambia – Needham et al. notes that on average, transportation costs account for a quarter of the non-medical expenditures ($25). Thus, we took a quarter of $25 and set that as the price per visit.

Data Extrapolation

We chose to transfer the estimated costs to other countries for which corresponding data are not available.

First, we used the classification system by the World Back to group all the counties by their regions.

Second, if we had transportation costs for any country within the region, then we assumed the same international dollar value of transportation costs between countries within the same region.

Next, if there were multiple countries within the given region, the closest country’s transportation cost in international dollars was used.

Finally, if there were no other countries in the region that use the same transportation, we found the nearest country with a known transportation cost.

The Estimated parameter values for each country are shown below.

| Country | Average transport cost  per travel (2005 I$)  (Costs transferred according to the  DCPP approach) |
| --- | --- |
| Afghanistan | 3.51 |
| Angola | 6.08 |
| Armenia | 8.14 |
| Azerbaijan | 8.14 |
| Bangladesh | 8.27 |
| Benin | 1.15 |
| Bhutan | 3.51 |
| Bolivia | 3.96 |
| Burkina Faso | 1.15 |
| Burundi | 6.08 |
| Cambodia | 8.14 |
| Cameroon | 1.15 |
| Central African Republic | 6.08 |
| Chad | 1.15 |
| Comoros | 2.11 |
| Congo | 6.08 |
| Cote d'Ivoire | 1.15 |
| Cuba | 3.96 |
| Democratic Republic of the Congo | 6.08 |
| Djibouti | 4.31 |
| Eritrea | 0.49 |
| Ethiopia | 0.49 |
| Georgia | 8.14 |
| Ghana | 1.15 |
| Guinea | 1.15 |
| Guinea-Bissau | 1.15 |
| Guyana | 3.96 |
| Haiti | 11.51 |
| Honduras | 3.96 |
| India | 3.51 |
| Indonesia | 5.44 |
| Kenya | 6.08 |
| Kiribati | 5.44 |
| Korea, Democratic Republic | 5.44 |
| Kyrgyzstan | 5.44 |
| Lao People Democratic Republic | 5.44 |
| Lesotho | 6.08 |
| Liberia | 1.15 |
| Madagascar | 2.11 |
| Malawi | 2.11 |
| Mali | 1.15 |
| Mauritania | 1.15 |
| Moldova | 8.14 |
| Mongolia | 5.44 |
| Mozambique | 2.11 |
| Myanmar | 5.44 |
| Nepal | 3.51 |
| Nicaragua | 3.96 |
| Niger | 1.15 |
| Nigeria | 1.15 |
| Pakistan | 3.51 |
| Papua New Guinea | 5.44 |
| Rwanda | 6.08 |
| Sao Thome | 6.08 |
| Senegal | 1.15 |
| Sierra Leone | 1.15 |
| Solomon Islands | 5.44 |
| Somalia | 6.08 |
| Sri Lanka | 0.52 |
| Sudan | 4.31 |
| Tajikistan | 8.14 |
| Tanzania | 6.08 |
| The Gambia | 2.08 |
| Timor Leste | 5.44 |
| Togo | 1.15 |
| Uganda | 4.3 |
| Ukraine | 8.14 |
| Uzbekistan | 8.14 |
| Viet Nam | 5.44 |
| Yemen | 4.31 |
| Zambia | 6.08 |
| Zimbabwe | 6.08 |

**References**

Ashworth A, Sultana K. Cost-effective treatment for severely malnourished children: what is the best approach? *Health Policy and Plann* 1997; 12(2):115-21.

Attanayake N, Fox-Rushby J, Mills A. Household costs of 'malaria' morbidity: a study in Matale district, Sri Lanka. *Trop Med Int Health* 2000; 5(9):595-606.

Buor D. Analysing the primacy of distance in the utilization of health services in the Ahafo-Ano South district, Ghana. *Int J Health Plann Mgmt* 2003; 18:293-311.

Deressa W, Hailemariam D, Ali A. Economic costs of epidemic malaria to households in rural Ethiopia. *Trop Med Int Health* 2007; 12(10):1148-56.

Gerstl S, Amsalu R, Ritmeijer K. Accessibility of diagnostic and treatment centres for visceral leishmaniasis in Gedaref State, northern Sudan. *Trop Med Int Health* 2006; 11(2):167-75.

Hussain H, Waters H, Khan AJ, Omer SB, Halsey NA. Economic analysis of childhood pneumonia in Northern Pakistan. *Health Policy and Planning* 2008; 23:438-42.

Kemp JR, Mann G, Simwaka BN, Salaniponi FML, Squire SB. Can Malawi's poor afford free tuberculosis services? *Bulletin of the World Health Organization* 2007; 85:580-5.

Mukherjee JS, Ivers L, Leandre F, Farmer P, Behforouz H. Antiretroviral Therapy in Resource-Poor Settings. *J Acquir Immune Defic Syndr* 2006; 43(1):s123-6.

Needham DM, Godfrey-Faussett P, Foster SD. Barriers to tuberculosis control in urban Zambia: the economic impact and burden on patients prior to diagnosis. *Int J Tuberc Lung Dis* 1998; 2(10):811-7.

Rheingans RD, Constenla D, Antil L, Innis BL, Bruer T. Economic and health burden of rotavirus gastroenteritis for the 2003 birth cohort in eight Latin American and Carribean countries. *Rev Panam Salud Publica* 2007; 21(4):192-204.

Wyss K, Kilima P, Lorenz N. Costs of tuberculosis for households and health care providers in Dar es Salaam, Tanzania. *Trop Med Int Health* 2001; 6(1):60-8.

Fox-Rushby JA, Foord F. Costs, effects and cost-effectiveness analysis of a mobile maternal health service in West Kiang, The Gambia. *Health Policy* 1996; 35:123-143.

Tuller DM, Bangsberg DR, Senkungu J, Ware NC, Emenyonu N, Weiser SD. Transportation Costs Impede Sustained Adherence and Access to HAART in a Clinic Population in Southwestern Uganda: A Qualitative Study. *AIDS Behav* 2009.

**Appendix 5. Budget impact analysis: Country-specific results**

In the present study, the financial requirements over a 10-year period for each of the GAVI-eligible countries are calculated based on county-specific scale-up scenarios and the GAVI’s co-financing scheme for new vaccines. Country-specific results not considering the savings (due to averted rotavirus disease burden) to the government are as follows:

| Countries | Total  (US$,  thous-ands) | Year 1 | Year 2 | Year 3 | Year 4 | Year 5 | Year 6 | Year 7 | Year 8 | Year 9 | Year10 |
| --- | --- | --- | --- | --- | --- | --- | --- | --- | --- | --- | --- |
|  |  | 2010 | 2011 | 2012 | 2013 | 2014 | 2015 | 2016 | 2017 | 2018 | 2019 |
| Afghanistan | 2,838 | 0 | 0 | 0 | 0 | 0 | 0 | 0 | 0 | 0 | 2,838 |
| Angola | 2,002 | 0 | 0 | 0 | 0 | 0 | 0 | 0 | 0 | 0 | 2,002 |
| Armenia | 2,046 | 146 | 176 | 202 | 224 | 217 | 213 | 212 | 214 | 218 | 223 |
| Azerbaijan | 8,893 | 913 | 924 | 909 | 880 | 852 | 840 | 849 | 873 | 908 | 945 |
| Bangladesh | 24,841 | 0 | 0 | 0 | 0 | 0 | 0 | 1,243 | 4,611 | 7,893 | 11,094 |
| Benin | 8,217 | 0 | 0 | 0 | 0 | 369 | 760 | 1,158 | 1,563 | 1,975 | 2,393 |
| Bhutan | 649 | 0 | 0 | 72 | 77 | 82 | 85 | 86 | 85 | 82 | 80 |
| Bolivia | 12,842 | 1,176 | 1,199 | 1,218 | 1,243 | 1,268 | 1,295 | 1,320 | 1,345 | 1,372 | 1,405 |
| Burkina Faso | 20,440 | 0 | 0 | 0 | 0 | 2,398 | 2,785 | 3,185 | 3,597 | 4,021 | 4,453 |
| Burundi | 2,190 | 0 | 0 | 0 | 0 | 0 | 0 | 0 | 0 | 0 | 2,190 |
| Cambodia | 10,230 | 0 | 0 | 0 | 0 | 1,198 | 1,405 | 1,609 | 1,811 | 2,007 | 2,199 |
| Cameroon | 27,221 | 0 | 0 | 2,197 | 2,594 | 3,018 | 3,445 | 3,879 | 3,954 | 4,027 | 4,107 |
| Central African Republic | 310 | 0 | 0 | 0 | 0 | 0 | 0 | 0 | 0 | 0 | 310 |
| Chad | 3,165 | 0 | 0 | 0 | 0 | 0 | 0 | 0 | 953 | 1,054 | 1,158 |
| Comoros | 341 | 0 | 0 | 0 | 0 | 0 | 0 | 57 | 76 | 95 | 114 |
| Congo | 311 | 0 | 0 | 0 | 0 | 0 | 0 | 0 | 0 | 0 | 311 |
| Cote d'Ivoire | 15,448 | 0 | 0 | 0 | 471 | 1,079 | 1,689 | 2,298 | 2,904 | 3,506 | 3,500 |
| Cuba | 10,801 | 980 | 1,002 | 1,045 | 1,100 | 1,129 | 1,143 | 1,138 | 1,117 | 1,088 | 1,059 |
| Democratic Republic of the Congo | 9,890 | 0 | 0 | 0 | 0 | 0 | 0 | 0 | 0 | 0 | 9,890 |
| Djibouti | 1,207 | 0 | 73 | 91 | 110 | 131 | 152 | 156 | 160 | 164 | 170 |
| Eritrea | 4,181 | 0 | 0 | 0 | 0 | 0 | 667 | 752 | 837 | 921 | 1,004 |
| Ethiopia | 53,578 | 0 | 0 | 0 | 0 | 0 | 7,684 | 9,185 | 10,703 | 12,233 | 13,773 |
| Georgia | 2,560 | 157 | 190 | 228 | 269 | 276 | 281 | 286 | 288 | 291 | 293 |
| Ghana | 18,817 | 0 | 0 | 0 | 0 | 0 | 3,624 | 3,700 | 3,768 | 3,832 | 3,893 |
| Guinea | 4,187 | 0 | 0 | 0 | 0 | 0 | 0 | 0 | 1,332 | 1,396 | 1,460 |
| Guinea-Bissau | 655 | 0 | 0 | 0 | 0 | 0 | 0 | 0 | 0 | 308 | 347 |
| Guyana | 738 | 63 | 66 | 67 | 70 | 72 | 75 | 77 | 80 | 83 | 85 |
| Haiti | 669 | 0 | 0 | 0 | 0 | 0 | 0 | 0 | 0 | 0 | 669 |
| Honduras | 13,185 | 1,132 | 1,268 | 1,282 | 1,295 | 1,309 | 1,327 | 1,348 | 1,376 | 1,407 | 1,441 |
| India | 725,123 | 0 | 0 | 9,361 | 39,584 | 69,474 | 99,007 | 128,172 | 127,382 | 126,539 | 125,604 |
| Indonesia | 122,263 | 0 | 0 | 0 | 11,515 | 13,550 | 15,615 | 17,689 | 19,754 | 21,908 | 22,233 |
| Kenya | 37,006 | 0 | 0 | 0 | 0 | 0 | 6,854 | 7,110 | 7,386 | 7,678 | 7,977 |
| Kiribati | 60 | 0 | 3 | 4 | 5 | 7 | 8 | 8 | 8 | 8 | 8 |
| Korea, Democratic Republic | 365 | 0 | 0 | 0 | 0 | 0 | 0 | 0 | 0 | 0 | 365 |
| Kyrgyzstan | 4,814 | 70 | 268 | 453 | 620 | 594 | 575 | 564 | 559 | 557 | 555 |
| Lao People Democratic Republic | 1,389 | 0 | 0 | 0 | 0 | 0 | 0 | 0 | 444 | 463 | 482 |
| Lesotho | 2,160 | 0 | 0 | 57 | 136 | 214 | 291 | 368 | 366 | 365 | 363 |
| Liberia | 6,410 | 0 | 0 | 0 | 0 | 688 | 830 | 979 | 1,136 | 1,302 | 1,474 |
| Madagascar | 16,264 | 0 | 0 | 0 | 0 | 0 | 2,719 | 2,982 | 3,250 | 3,520 | 3,793 |
| Malawi | 17,240 | 0 | 0 | 0 | 0 | 2,286 | 2,519 | 2,753 | 2,989 | 3,227 | 3,465 |
| Mali | 19,726 | 0 | 0 | 0 | 0 | 2,219 | 2,614 | 3,033 | 3,479 | 3,949 | 4,432 |
| Mauritania | 729 | 0 | 0 | 0 | 0 | 0 | 0 | 0 | 196 | 243 | 290 |
| Moldova | 2,482 | 216 | 230 | 245 | 261 | 262 | 262 | 259 | 255 | 249 | 242 |
| Mongolia | 2,563 | 0 | 267 | 275 | 285 | 293 | 298 | 296 | 291 | 283 | 275 |
| Mozambique | 5,747 | 0 | 0 | 0 | 0 | 0 | 0 | 0 | 1,396 | 1,915 | 2,436 |
| Myanmar | 4,622 | 0 | 0 | 0 | 0 | 0 | 0 | 338 | 879 | 1,427 | 1,979 |
| Nepal | 4,348 | 0 | 0 | 0 | 0 | 0 | 0 | 98 | 752 | 1,415 | 2,083 |
| Nicaragua | 8,564 | 886 | 884 | 876 | 866 | 855 | 847 | 842 | 838 | 836 | 834 |
| Niger | 3,505 | 0 | 0 | 0 | 0 | 0 | 0 | 0 | 0 | 1,458 | 2,048 |
| Nigeria | 136,236 | 0 | 0 | 0 | 15,767 | 17,096 | 18,407 | 19,690 | 20,946 | 22,178 | 22,153 |
| Pakistan | 134,794 | 0 | 0 | 0 | 17,901 | 18,220 | 18,628 | 19,168 | 19,802 | 20,489 | 20,586 |
| Papua New Guinea | 4,759 | 0 | 0 | 0 | 719 | 707 | 694 | 679 | 663 | 647 | 650 |
| Rwanda | 15,343 | 0 | 0 | 0 | 0 | 2,410 | 2,469 | 2,529 | 2,590 | 2,648 | 2,697 |
| Sao Thome | 229 | 0 | 0 | 15 | 20 | 25 | 30 | 35 | 35 | 35 | 35 |
| Senegal | 10,148 | 0 | 0 | 0 | 0 | 0 | 1,612 | 1,820 | 2,029 | 2,238 | 2,449 |
| Sierra Leone | 4,180 | 0 | 0 | 0 | 0 | 0 | 393 | 609 | 830 | 1,057 | 1,290 |
| Solomon Islands | 639 | 0 | 52 | 58 | 65 | 71 | 78 | 78 | 78 | 79 | 79 |
| Somalia | 994 | 0 | 0 | 0 | 0 | 0 | 0 | 0 | 0 | 496 | 498 |
| Sri Lanka | 13,622 | 0 | 0 | 1,109 | 1,316 | 1,532 | 1,749 | 1,958 | 1,974 | 1,985 | 1,998 |
| Sudan | 45,092 | 0 | 0 | 1,739 | 3,137 | 4,533 | 5,940 | 7,368 | 7,409 | 7,459 | 7,507 |
| Tajikistan | 7,894 | 383 | 544 | 710 | 882 | 893 | 900 | 902 | 900 | 894 | 886 |
| Tanzania | 30,166 | 0 | 0 | 0 | 0 | 0 | 0 | 7,268 | 7,436 | 7,634 | 7,828 |
| The Gambia | 1,826 | 0 | 0 | 0 | 0 | 279 | 289 | 299 | 309 | 320 | 330 |
| Timor Leste | 1,889 | 0 | 0 | 0 | 212 | 227 | 244 | 265 | 291 | 320 | 330 |
| Togo | 5,437 | 0 | 0 | 0 | 0 | 0 | 821 | 954 | 1,087 | 1,221 | 1,355 |
| Uganda | 11,912 | 0 | 0 | 0 | 0 | 0 | 0 | 0 | 3,015 | 3,961 | 4,936 |
| Ukraine | 18,064 | 104 | 840 | 1,551 | 2,233 | 2,197 | 2,180 | 2,184 | 2,213 | 2,257 | 2,304 |
| Uzbekistan | 27,559 | 174 | 1,245 | 2,334 | 3,454 | 3,485 | 3,485 | 3,450 | 3,390 | 3,312 | 3,230 |
| Viet Nam | 48,036 | 0 | 0 | 0 | 0 | 7,368 | 7,707 | 7,969 | 8,172 | 8,332 | 8,487 |
| Yemen | 12,256 | 0 | 0 | 0 | 0 | 0 | 534 | 1,474 | 2,434 | 3,412 | 4,403 |
| Zambia | 18,123 | 0 | 0 | 0 | 0 | 2,719 | 2,843 | 2,964 | 3,083 | 3,199 | 3,314 |
| Zimbabwe | 2,922 | 0 | 0 | 0 | 0 | 0 | 0 | 0 | 442 | 976 | 1,504 |
